# Exploring Inertial-Based Wearable Technologies for Objective Monitoring in Sports-Related Concussion: A Single-Participant Report

**DOI:** 10.1093/ptj/pzac016

**Published:** 2022-02-23

**Authors:** Dylan Powell, Samuel Stuart, Alan Godfrey

**Affiliations:** Department of Computer and Information Sciences, Northumbria University, Newcastle-upon-Tyne, United Kingdom; Department of Sport, Exercise and Rehabilitation, Northumbria University, Newcastle-upon-Tyne, United Kingdom; Department of Computer and Information Sciences, Northumbria University, Newcastle-upon-Tyne, United Kingdom

**Keywords:** Brain Concussion, Return to Play, Rugby, Sports Medicine, Wearable Technologies

## Abstract

**Objective:**

Challenges remain in sports-related concussion (SRC) assessment to better inform return to play. Reliance on self-reported symptoms within the Sports Concussion Assessment Tool means that there are limited data on the effectiveness of novel methods to assess a player’s readiness to return to play. Digital methods such as wearable technologies may augment traditional SRC assessment and improve objectivity in making decisions regarding return to play.

**Methods:**

The participant was a male university athlete who had a recent history of SRC. The single-participant design consisted of baseline laboratory testing immediately after SRC, free-living monitoring, and follow-up supervised testing after 2 months. The primary outcome measures were from traditional assessment (eg, Sports Concussion Assessment Tool and 2-minute instrumented walk/gait test; secondary outcome measures were from remote (free-living) assessment with a single wearable inertial measurement unit (eg, for gait and sleep).

**Results:**

The university athlete (age = 20 years, height = 175 cm, weight = 77 kg [176.37 lb]) recovered and returned to play 20 days after SRC. Primary measures returned to baseline levels after 12 days. However, supervised (laboratory-based) wearable device assessment showed that gait impairments (increased step time) remained even after the athlete was cleared for return to play (2 months). Similarly, a 24-hour remote gait assessment showed changes in step time, step time variability, and step time asymmetry immediately after SRC and at return to play (1 month after SRC). Remote sleep analysis showed differences in sleep quality and disturbance (increased movement between immediately after SRC and once the athlete had returned to play [1 month after SRC]).

**Conclusion:**

The concern about missed or delayed SRC diagnosis is growing, but methods to objectively monitor return to play after concussion are still lacking. This report showed that wearable device assessment offers additional objective data for use in monitoring players who have SRC. This work could better inform SRC assessment and return-to-play protocols.

**Impact:**

Digital technologies such as wearable technologies can yield additional data that traditional self-report approaches cannot. Combining data from nondigital (traditional) and digital (wearable) methods may augment SRC assessment for improved return-to-play decisions.

**Lay Summary:**

Inertia-based wearable technologies (eg, accelerometers) may be useful to help augment traditional, self-report approaches to sports-related concussion assessment and management by better informing return-to-play protocols.

## Introduction

In the United Kingdom, more than 1 million people per year attend accident and emergency departments because of head injuries.^1^ Severity of head injuries can range from mild traumatic brain injury (mTBI, ie, concussion), which often does not require hospital admission, to serious (TBI), which can require hospital admission and surgical intervention. An increasing incidence of mTBI has been attributed to participation in sports, such as rugby,^2^^,^^3^ and is referred to as sports-related concussion (SRC). Several impairments characterize SRC, including physical function (balance and walking), psychological, sleep quality, and symptoms.^3^ At present, there is no gold standard for diagnosing or monitoring recovery from SRC.^4^ Consequently, there is concern about the long-term impact of premature return to play or habitual activity before full recovery.^5–7^ Here, return to play is defined as clearance by a medical professional to return to contact training/play.

A common method of SRC assessment is the fifth version of the (pen- and paper-based) Sports Concussion Assessment Tool (SCAT5). It assesses symptom severity through the Post-concussion Symptom Severity Scale, cognition via the Standardized Assessment of Concussion, and physical function by using the modified Balance Error Scoring System and examining tandem gait. The implementation and widespread uptake of SCAT5 has (somewhat) helped standardize concussion recognition and reporting across many sports. However, an unwanted consequence of that is generalizability—namely, the adoption of a one-size-fits-all approach for assessment and return to play with the use of the SCAT5 only.^8^ Because of the SRC response and recovery heterogeneity, return-to-play periods of mandatory rest and rehabilitation are guidelines (generalized and nonspecific), making it difficult to know when it is safe for an individual who may take longer to recover to return to play.^9^^,^^10^

Furthermore, the SCAT5 is a snapshot assessment tool, confounded by subjective approaches of manual rating and self-reporting during each subcomponent/test.^11–13^ The fact that the SCAT5 gives little weighting to other impairments in SRC diagnosis limits data integration and multimodal assessment. For example, sleep quality is superficially and subjectively assessed by the SCAT5 despite its prevalence, whereas up to 70% of patients with TBI experience sleep disturbance.^14^^,^^15^^,^^16^ Thus, the SCAT5 can only aid in an SRC diagnosis, and clinical judgment often must be used,^3^ making it extremely challenging to accurately/objectively and transparently monitor and assess SRC impairments. This situation justifies the demand for new, objective methods to better track SRC recovery and better inform return to play.

Recently, digital technologies were suggested to continuously and objectively measure outcomes; such as approach could augment snapshot SRC assessment.^17^^,^^18^ Additionally, remote monitoring (to overcome snapshot assessments) is more feasible and desirable because of the widespread availability of wearable digital technologies and other associated technologies (eg, smartphones), which can gather high-resolution data continuously for many days.^19^ Prevalent wearable examples are found within older adult neurological cohorts and in the use of inertial measurement units (IMUs, ie, accelerometer and/or gyroscope-based wearable technologies) to estimate broad physical activity or sleep behaviors as well as fine motor performance, such as balance and gait, from supervised and remote assessments.^20–25^ To our knowledge, no study has examined the use of wearable IMUs for holistic SRC assessment. Using these digital technologies would enable high-resolution data capture to augment snapshot assessment as well as remote monitoring to assess habitual SRC impairments over longitudinal periods. In short, using digital approaches to continuously monitor SRC impairments (eg, sleep and gait) may enhance current methods and deliver more objective and personalized SRC assessment digital (bio)markers.

The purpose of this single-participant report/study was to explore the feasibility and potential of a wearable IMU to improve SRC assessment in a rugby player and monitoring by augmenting traditional approaches. The study participant completed traditional supervised/reference testing (SCAT5), yielding primary outcomes. The participant also undertook supervised instrumented and remote monitoring, yielding secondary (digital) outcomes (gait and sleep) before and after SRC. We hypothesized that using supervised and remote (free-living) assessments with a wearable IMU would yield greater insights than traditional SRC assessment alone.

## Methods

### Participant Recruitment

The research team is conducting a large cohort study focusing on multimodal data collection within university rugby teams during 2019 to 2022; players are assessed longitudinally for SRC. For the larger cohort study, inclusion criteria were as follows: ≥18 years of age, English as a first language, and no impairment that would prohibit participants from safely performing the supervised tasks or remote assessments. Ethics consent for the project was granted by the Northumbria University Ethics Committee (reference no. 3672). The participant was given an information sheet that detailed the study, and the participant gave informed written consent prior to testing. All laboratory testing took place at Northumbria University Sport Central (Newcastle-upon-Tyne, UK) during the 2019 to 2020 season.

A player who sustained an SRC (age = 20 years, height = 175 cm, weight = 77 kg) was chosen at random from the larger pool of players for assessment. This player had been followed-up within 24 hours after sustaining an SRC. At the time of testing, he had completed 16 years of full-time education and self-reported a total of 5 head injuries, with his most recent prior to testing occurring in spring 2019. University medical records detailed that his recovery time from a previous concussion was 19 days.

### Technology

The IMU-based wearable device assessment was conducted using the low-cost AX6, which contains accelerometer and gyroscope sensors (Axivity, Newcastle-upon-Tyne, UK; https://axivity.com; 2.3 × 3.3 × 0.8 cm; 11 g). For the purposes of this study, accelerometer data only are presented. Throughout testing, the sampling rate (number of data points per second) for the AX6 was set to 100 Hz. The participant wore the AX6 on the lower back (fifth lumbar vertebra [L5]); it was attached with double-sided tape and an extra bandage (Hypafix; BSN Medical Ltd, Hull, UK) to maintain position. L5 was chosen on the basis of IMU signal-processing algorithms previously defined and validated to quantify supervised and remote gait.^26^^,^^27^ The participant wore the AX6 continuously for the duration of supervised and remote assessments (the latter comprising 24 hours at each time point).

### Design Phases: Experimental Protocol

The participant was assessed before concussion, after concussion, and after return to play using both traditional (SCAT5) and wearable device assessments as described below.

#### Primary Outcome: SCAT5

The SCAT5 was completed by a physical therapist at baseline (conducted in 2018 as part of a routine university player health assessment but not by a member of the research team) as well as 1 hour, 2 days, 5 days, 12 days, and approximately 2.5 months after SRC.

#### Secondary Outcome: Wearable IMU

Data were processed using custom-made and validated MATLAB (The MathWorks, Inc, Natick, MA, USA) algorithms to estimate supervised and remote gait characteristics.^27^^,^^28^ Raw IMU data were examined before SRC, after SRC (1 day), and after return to play (28 days after SRC) as follows.

For the supervised (laboratory) gait assessment, the participant walked continuously and as fast as he could (without running) back and forth around cones (10 m apart) as part of the 2-minute walk test.^29^^,^^30^ Timing by the wearable device began on the first step. Recording ended after the participant completed the walk (manual, by stopwatch) or after the last purposeful footfall (as detected by the wearable device). The latter was determined from the vertical acceleration exceeding a predetermined threshold.^31^

To evaluate gait characteristics, raw IMU accelerometer data were used to identify the initial contact and final contact times within the gait cycle.^32^ Initial contact and final contact estimation facilitated quantification of step time (in seconds); a walking bout was previously defined as ≥3 steps with consecutive steps within a 0.25- to 2.25-second window.^33^ Subsequently, variability and asymmetry gait characteristics were quantified from alternate values within an array of step times. Here, we examined all gait data (from gait initiation to termination). For exploratory purposes, we quantified 2 variations of variability that are common in the literature (one may provide greater insights than the other), as follows. In the variability approach, the combined SD for left and right steps was calculated by taking the square root of the mean variance of the left and right steps. This method avoids confounding step-to-step variability with variation originating from asymmetry between left and right steps.^34^ Here, true left and right footsteps were not explicitly identified, so alternate values were chosen. In the SD approach, the SD of all steps was calculated for left and right—that is, left and right were not separated as alternating values. Asymmetry was calculated as the absolute difference between left and right steps. Again, true left and right footsteps were not explicitly identified, so alternate values were chosen.

For remote gait assessment (immediately after SRC, 1 month after SRC, and after return to play), the wearable IMU was worn continuously for 24 hours while the participant was asked to perform his normal/habitual routine. A validated algorithm^27^ quantified gait/walking bouts; for the purposes of this study, gait bouts of ≥120 seconds/2 minutes were examined. Once possible gait bouts were detected, the same gait characteristics were calculated as for the supervised assessment (described above).

For sleep assessment (immediately after SRC, 1 month after SRC, and after return to play), the wearable IMU was able to record whole-body movement based on the L5 attachment (and proximity to the wearer’s center of mass). Here, we broadly examined accelerometer data to provide insights on nocturnal activity to evaluate sleep quality.

### Role of the Funding Source

The funders played no role in the design, conduct, or reporting of this study.

## Results

### Primary Outcome

**Figure 1:**
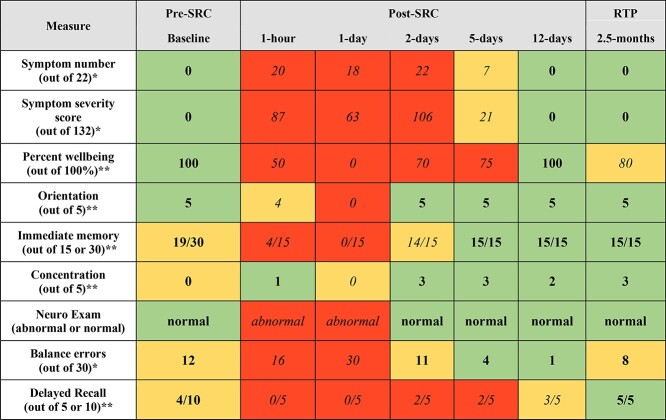
Use of the fifth version of the Sports Concussion Assessment Tool (SCAT5) before sports-related concussion (SRC), immediately after SRC, and >2 months later. Green/bold data indicate that the player returned to baseline levels. Yellow (bold or italic) data indicate abnormal findings. Red/italic data indicate that the player scored worse than at baseline. Assessments before and after SRC were performed by different physical therapists. ^*^A lower score was better (more normal). ^*^^*^A higher score was better (more normal). Neuro Exam = neurological examination; RTP = returned to play.

Post-SRC symptom severity data showed a sharp increase in the symptom score 1 hour after injury (score of 87), peaking at day 2 after injury (score of 106) before decreasing and returning to normal at day 12 (score of 0) (Fig. 1). Balance errors also peaked (score of 11) shortly after injury before gradually decreasing 12 days after injury. Similarly, immediate memory returned to normal function 5 days after injury. The participant was cleared to return to play and contact sport by an independent general practitioner after 20 days. At reassessment (2.5 months after injury), there was a decline in some SCAT5 outcomes (decreased well-being and increased balance errors). For the purposes of this case study, Fig. 1 displays a subjective and unvalidated color coding schema to infer player SCAT5 scores.

### Secondary Outcomes

#### Supervised Gait (2-Minute Walk Test)

**Figure 2:**
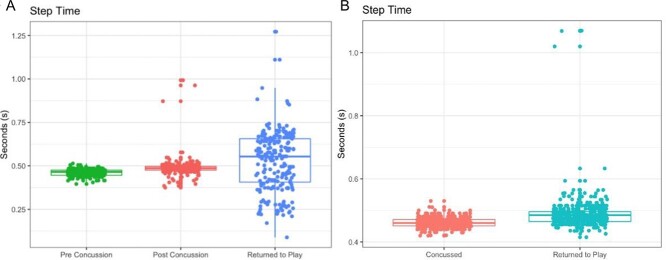
Gait assessment. (A) Supervised (laboratory) gait assessment (2-minute walk test) for step time before sports-related concussion (SRC), after SRC, and after return to play. (B) Remote gait assessment for step time.

Wearable IMU assessment was well tolerated by the participant, and no concerns of discomfort were raised. There were differences for gait before SRC, immediately after SRC, and at the 2.5-month follow-up (Fig. 2). The results showed increases in step time (0.461, 0.491, and 0.529 seconds), step time variability (0.018, 0.151, and 0.255 seconds), and step asymmetry (0.012, 0.003, and 0.036 seconds) for baseline, after concussion, and at the 2-month follow-up, respectively (Tab. 1).

**Table 1:** Gait Characteristics From Supervised 2-Minute Walk (Initiation to Termination)^a^

**Gait Parameter**	**Before SRC**	**1 d After SRC**	**2.5 mo After SRC**
Step time, s	0.461	0.491	0.529
Step time variability (Var), s	0.018	0.151	0.255
Step time variability (SD), s	0.019	0.057	0.171
Step time asymmetry, s	0.012	0.003	0.036

^a^ SD = standard deviation approach; SRC = sports-related concussion; Var = variability approach.

#### Remote Gait

Remote wearable IMU gait assessment showed 4 bouts of (sustained) walking for ≥2 minutes immediately after SRC and 6 bouts 1 month after SRC (Tab. 2). Mean step times were shorter immediately after SRC than 1 month later. There were no clearly observable trends for step time variability between different time points, but what was observed during gait assessment 1 month after SRC was the difference in variability outcomes for contrasting calculation methods. Asymmetry was notably higher 1 month after SRC; this finding may have been influenced by some abnormal outliers (Fig. 2). (Additional data are presented in supplementary material for further gait characteristic exploration; see Supplementary Data.

**Table 2:** Remote Gait From Bouts of Walking for ≥120 Seconds/2 Minutes (Initiation to Termination)^a^

**Gait Parameter**	**Value at Indicated Time**					
1 d after SRC	2:43 pm	2:51 pm	4:33 pm	4:36 pm		
Step time, s	0.464	0.458	0.462	0.440		
Step time variability (Var), s	0.016	0.013	0.041	0.043		
Step time variability (SD), s	0.016	0.013	0.041	0.043		
Step time asymmetry, s	0.005	0.001	0.004	0.004		
1 mo after SRC	11:47 am	11:50 am	12:38 pm	12:42 pm	12:54 pm	1:01 pm
Step time, s	0.508	0.504	0.486	0.490	0.491	0.489
Step time variability (Var), s	0.028	0.025	0.017	0.021	0.016	0.057
Step time variability (SD), s	0.033	0.031	0.020	0.024	0.022	0.058
Step time asymmetry,s	0.034	0.036	0.022	0.024	0.031	0.008

^a^ SD = standard deviation approach; SRC = sports-related concussion; Var = variability approach.

#### Sleep Assessment

**Figure 3:**
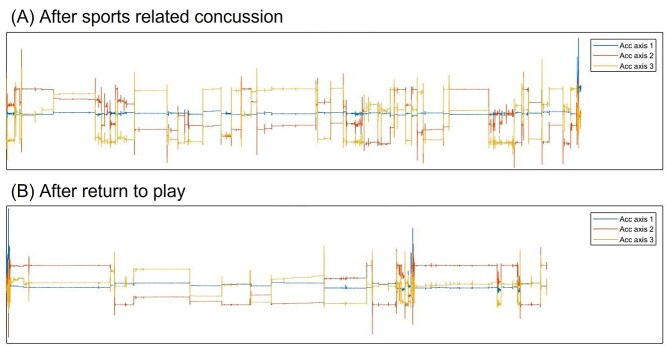
Sleep analysis (general nocturnal movement for 6 h) after sports-related concussion (A) and after return to play (B).

Raw IMU accelerometer data were used to broadly examine and compare nocturnal/sleep activity at different times. Fig. 3 shows notable differences between time points, with greater frequency of movement as defined by the changing orientation of the accelerometer signal (eg, axis 3, representing the transverse plane about the longitudinal axis within the IMU). This high-level visual examination suggested that the participant had poorer sleep quality because of unsettled behavior and changing from lying on right to supine to left postures (Fig. 3A). In contrast, Figure 3B shows far fewer periods of movement and longer periods of less sleep disturbance, potentially indicating improved sleep quality 1 month after SRC.

**Figure 4:**
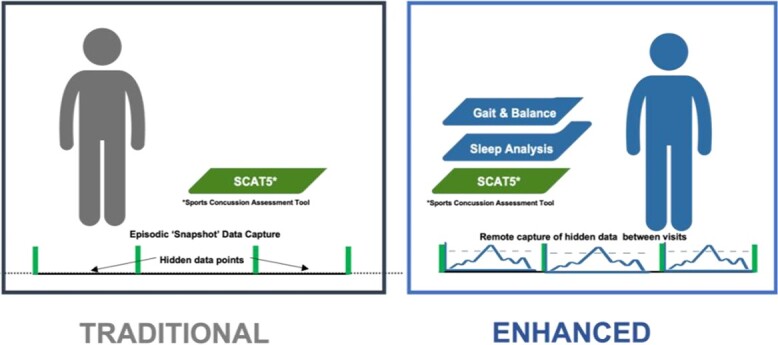
Traditional (fifth version of the Sports Concussion Assessment Tool) vs enhanced assessment for return to play, with additional metrics, such as gait, balance, and sleep analysis.

## Discussion

The purpose of this single-participant report/study was to examine the feasibility and potential of supervised and remote assessments with a wearable IMU to augment traditional SRC assessment. To our knowledge, this is the first study to deploy an IMU-based wearable device for SRC monitoring during a return-to-play protocol. The use of IMU-based wearable technologies can yield a larger number of digital (bio)marker characteristics than the use of traditional assessments, such as temporal gait characteristics and trends on sleep quality (Fig. 4). Free-living/remote assessment of gait and sleep with an IMU was well tolerated and may be particularly useful when examining individuals with complex medical and frequent SRC histories. Continuous monitoring with wearable technologies over long periods may be required to detect more sensitive or subtle deficits that become more evident beyond supervised assessment or persist after return to play.

### Enhancing SRC Assessment

Our results reinforce the challenges of traditional approaches with the SCAT5, which may not be sensitive (significant ceiling effects) to the varied recovery participants experience after SRC or useful in dealing with complex recovery trends. Additionally, the fact that different clinicians may administer/record the SCAT5 at various time points can contribute to variations. For example, the choice of cognitive test difficulty (5- or 10-word list) can affect reliability or consistency in testing, meaning that some players are being tested on different difficulty ratings/scoring. This situation is evident here (Fig. 1) in that the participant was scored on the 10-word list at baseline because SCAT data were routinely collected as part of university player welfare but not as part of the present study. For the protocol adopted as part of the larger study, participants were scored on 5-word lists after injury, making comparisons between scores more challenging.

As shown in Figure 1, SCAT5 outcomes quickly trended to baseline after 12 days. Specifically, full symptom resolution and improvement in both cognitive and balance scores were seen (Fig. 1), indicating progress in recovery. A general practitioner approved return to play (full contact rugby) by day 20 on the basis of these SCAT5 outcomes and (subjective) clinical judgment. The decline in some SCAT5 outcomes (decreased well-being and increased balance errors) 2.5 months after injury is difficult to attribute to a specific injury or time point. However, it is reasonable to speculate that incomplete recovery from initial injury and/or playing through a new injury contributed to the decline in outcomes. These data highlight how challenging it can be to rely on subjective outcomes to assess players’ injury/recovery status.

The addition of a wearable IMU–facilitated instrumented assessment was an efficient method for gaining a more representative and holistic impression of SRC impact and recovery. Data from the wearable IMU assessment allowed investigation into more detailed trends for gait characteristics longitudinally (during recovery and 1 month after SRC). Specifically, within the supervised wearable IMU gait assessment, there were differences for step time immediately after SRC, consistent with impaired motor control.^7^ Unsupervised/remote wearable IMU gait assessment suggested gait impairments even on return to play; these data could further support an incomplete/full recovery.^35^

Wearable IMU assessment afforded the opportunity to obtain high-resolution clinical gait characteristics, such as mean step time and variations (variability and asymmetry)—something that is not possible with traditional approaches (SCAT5). Our exploratory analysis suggested that these data may be clinically useful but that it is also important to consider how those outcomes are calculated and presented and in what environment. For example, notable differences were observed for different variability outcomes in the laboratory (Tab. 1) and free-living situation (Tab. 2). Here, the 2 common methods used to estimate step time variability resulted in differences in outcomes even during the same walking bouts in supervised or remote assessment. Step time was calculated as the difference between the estimation of initial contact and the estimation of final contact within the gait cycle, as previously described.^28^^,^^32^^,^^36^ Step time variability (SDs of left and right steps) was estimated via the square root of the mean variance of the left and right steps, and step time asymmetry was calculated from the difference between right and left steps (mean values).^28^ Increased step time variability and asymmetry have been closely linked with impaired or dysfunctional motor control and deficits in similar groups, such as people with mTBI and TBI.^37^

We were able to observe broad changes and trends in step time in people with other conditions, such as Parkinson disease and chronic mTBI.^22^^,^^38^^,^^39^ However, at present, there is a lack of consensus about exactly what constitutes normal or impaired gait, particularly in people with SRC/mTBI.^40^ Therefore, we were unable to provide exact values or be precise about an agreed meaningful clinical change but will explore these topics as part of our ongoing work. Furthermore, this case study cannot be directly compared with studies examining larger cohorts of participants with TBI, which observed differences for step time variability before SRC and after SRC. We also found large step asymmetry 1 month after SRC, which may be explained by outliers at that time point. However, recent research^41^ suggests step time asymmetry could also be linked to energy demand and compensatory behaviors due to the energy cost of impaired gait.^41^^,^^42^ Our results would align with that research, because step time asymmetry decreased immediately after SRC and then increased 1 month after SRC, once the participant had returned to play.

Overall, the addition of a wearable IMU–facilitated instrumented assessment was an efficient method for gaining a more representative and holistic impression of gait trends and recovery. However, collecting data on more participants is required for robust investigation and interpretation of step time asymmetry during SRC recovery.

### Remote Assessment for Enhancing Return-to-Play Decisions

Traditionally, return-to-play clearance and readiness to return to play are within the realm of general practitioners or medical professionals who use clinical judgment to form the basis of their opinions. They may use tools such as the SCAT5 to aid in their judgment but cannot use this approach in isolation. Furthermore, symptom resolution is the main driver in return to play,^43^ with full resolution and successful navigation of graduated return to play being necessary precursors before return to play.

Remote assessment using a wearable IMU is a unique example of an objective digital method that has been successfully tested in people with other neurological conditions, such as Parkinson disease.^44^^,^^45^ Remote assessment affords the opportunity for habitual monitoring of a variety of individualized digital (bio)makers, such as gait and sleep.^46^ The adoption of such technology can improve episodic data points (eg, supervised data collection), enabling a more holistic and in-depth SRC assessment. Remote gait assessment may be more clinically useful and holistic than isolated supervised assessment (detailed below) when comparing post-SRC recovery and return to play.

Alongside traditional return to play, the general practitioner or clinician would have additional objective data to aid in return-to-play decisions. Instead of the number of balance errors from the SCAT5, the 7-day average in gait variability and asymmetry could be presented and compared with normative and/or baseline performance. Overall, a wealth of additional variables could be used. Here, preliminary wearable-based gait characteristics suggest some insight to motor impairment during return to play, highlighting an incomplete recovery of the participant. However, because this was a case study, strong clinical conclusions cannot be drawn, but the potential value and benefits of remote gait assessment should be considered and explored further, such as examining compensatory gait strategies that may be evident in those with frequent SRC histories.

### Sleep Analysis

Recent research has indicated that subtypes of post-concussion presentation may be specifically linked to sleep disturbance.^47^ The wearable technologies used here were able to monitor generic movement patterns (ie, nocturnal sleep activity), which were used to estimate sleep quality (Fig. 3). There were visual differences between sleep analysis time points, with increased sleep disturbances immediately after SRC compared with 1 month after SRC and return to play. Although a detailed evaluation of sleep metrics was outside the scope of the present study, wearable technologies may be able to provide additional nocturnal data for inference of sleep quality and monitoring. For example, there are alternative wearable IMU-based algorithms for a more robust sleep analysis.^48^ However, the referenced study used a specific methodology developed for use with a wrist-worn IMU but alluded to what is achievable. Future studies may consider the use of different/multiple wearable technologies for day and night/sleep assessments, although that approach would increase the complexity of data synchronization and processing.

### Limitations

The limitations of single-participant research include the generalizability of study conclusions. However, this single-participant research study demonstrated the potential for a wearable IMU-based assessment to provide objective gait characteristics during supervised and remote assessments at different time points. The IMU also provided some insight for possible sleep disturbances. However, several extraneous variables should be considered in an SRC analysis. First, the fact that no baseline assessment of previous injuries was conducted may have affected gait patterns and thus data captured by the IMU. Second, the present study did not include the use of any self-reported sleep diary, which was previously used in sleep activity monitoring to better contextualize IMU data^48^^,^^49^ and which should be included in future studies. Finally, the processing of IMU data requires specialized training and prior experience.^50^^,^^51^ Therefore, a barrier to easily capturing and interpreting IMU data for clinical deployment is the development of “no-code” software that clinicians or non-technically skilled researchers can easily use.^51^ To improve accessibility and transparency of data collection methodologies, future research should also focus on optimizing open-source approaches for physical activity and sleep detection, as proposed for waist-worn sensors.^52^

Finally, the context in which the remote assessment was conducted remained unknown. These unknown factors and contexts (eg, types of terrain encountered in the walking environment) were heterogeneous in how they affected the results.^53^ In addition, it is not possible to quantify whether there are different free-living mobility habits among populations or whether people display any compensatory behavior strategies (eg, refraining from talking or performing other tasks while walking) that may further affect results. These may explain why there are differences between supervised and unsupervised wearable device assessments. Future research should investigate the contexts of free-living assessments, as previously described.^54^

### Toward Objective Multimodal Assessment

Pervasive SRC assessment techniques, such as the SCAT5, that collect self-reported/subjective measures are restricted to highly controlled supervised environments, such as physical therapy clinics or health centers. Although useful data are collected, these assessments are unable to capture habitual behaviors/trends and therefore omit a wealth of data that could be captured by nonobtrusive digital approaches. This idea is supported by the Concussion Consensus Statement, which highlighted that digital objective approaches are crucial to improving the ability to diagnose and monitor SRC impairments.^43^ Therefore, harnessing objective digital approaches such as wearable technologies is key to advancing diagnostics and precision in SRC assessment and monitoring. The Concussion Consensus Statement^43^ also stated that monitoring single isolated impairments is unlikely to drive any step changes in SRC assessment and diagnosis. Therefore, a necessary step change and progression in SRC assessment will require combining complementary multimodal technologies and moving away from reliance on the SCAT5, which collects data on isolated impairments. Collecting and integrating multimodal digital approaches (for symptom, visual, cognitive, and free-living motor and sleep analysis) will likely augment and enhance data gathered from traditional methods.

There is growing concern about inappropriate management of SRC in contact sports and premature return to play. This situation is complicated by a lack of methods to objectively monitor players during their recovery and return to play. This single-participant report/study showed that wearable device assessment can be well tolerated and yields objective outcomes to monitor players who have SRC. However, the precise SRC impairments that should be measured with IMUs is still unclear and will require work with more individuals. Combining data from both nondigital (traditional) and digital technologies can inform a multimodal approach that may deliver more comprehensive SRC assessments and improved return-to-play decisions.

## Supplementary Material

Supplementary_data_pzac016
