# Supplementary material for: Exploring Inertial-Based Wearable Technologies for Objective Monitoring in Sports-Related Concussion: A Single-Participant Report
Source: Phys Ther. 2022 Feb 23;102(5):pzac016. doi: 10.1093/ptj/pzac016 (PMC9155164; doi:10.1093/ptj/pzac016)
Supplement: Supplementary_data_pzac016 [file supplementary_data_pzac016.pdf]

Supplementary data

Here are calculations for exploratory purposes that readers may find interesting. Data below shows the calculations of step time, step time variability:Var (the square root of the mean variance of the left and right steps), step time variability: SD (the standard deviation of all steps where calculated for left and right steps) and step time asymmetry (absolute difference between left and right steps) for supervised (1A) and remote assessment (1B).

Data is presented for all gait data **(initiation to termination)** and steady state, whereby the latter removes gait initiation and termination i.e., the removal of the initial 10 steps and final 10 steps of the total walking bout. Here we showcase all gait bout data compared to steady state to highlight data analysis possibilities are arising difference in gait characteristics that may emerge.

Appendix 1A (Supervised assessment)

		Pre-SRC (s)	Post-SRC (s)	1 month post SRC (s)
**All gait data**	Step time	0.461	0.491	0.529
	Step time variability (Var)	0.018	0.151	0.255
	Step time variability (SD)	0.019	0.057	0.171
	Step time asymmetry	0.012	0.003	0.036
**Steady state**	Step time	0.461	0.492	0.532
	Step time variability (Var)	0.018	0.058	0.170
	Step time variability (SD)	0.019	0.058	0.171
	Step time asymmetry	0.011	0.002	0.041

Var = Variability. SD = Standard deviation

Appendix 1B (Remote assessment))

Post-SRC (1-day)								
		**Bout 1**	**Bout 2**	**Bout 3**	**Bout 4**	**Bout 5**	**Bout 6**	**Mean**
**All gait data**	Step time (s,seconds)	0.464	0.458	0.462	0.440	--	--	0.456
	Step time variability (Var)	0.016	0.013	0.041	0.043	--	--	0.028
	Step time variability (SD)	0.016	0.013	0.041	0.043	--	--	0.028
	Step time asymmetry	0.005	0.001	0.004	0.004	--	--	0.004
**Steady state**	Step time (s,seconds)	0.463	0.457	0.461	0.434	--	--	0.454
	Step time variability (Var)	0.015	0.013	0.042	0.050	--	--	0.030
	Step time variability (SD)	0.015	0.013	0.042	0.050	--	--	0.030
	Step time asymmetry	0.005	0.000	0.004	0.006	--	--	0.004
**Once Returned to Play (1-month)**								**Mean**
**All gait data**	Step time (s,seconds)	0.508	0.504	0.486	0.490	0.491	0.489	0.497
	Step time variability (Var)	0.028	0.025	0.017	0.021	0.016	0.057	0.023
	Step time variability (SD)	0.033	0.031	0.020	0.024	0.022	0.058	0.027
	Step time asymmetry	0.034	0.036	0.022	0.024	0.031	0.008	0.029
**Steady state**	Step time (s,seconds)	0.512	0.503	0.468	0.489	0.492	0.493	0.493
	Step time variability (Var)	0.026	0.029	0.064	0.094	0.016	0.069	0.053
	Step time variability (SD)	0.031	0.033	0.020	0.024	0.023	0.069	0.027
	Step time asymmetry	0.032	0.028	0.021	0.019	0.033	0.001	0.025

Var = Variability. SD = Standard deviation
